# Continuously elevated serum matrix metalloproteinase-3 for 3 ~ 6 months predict one-year radiographic progression in rheumatoid arthritis: a prospective cohort study

**DOI:** 10.1186/s13075-015-0803-2

**Published:** 2015-10-14

**Authors:** Jian-Da Ma, Xiu-Ning Wei, Dong-Hui Zheng, Ying-Qian Mo, Le-Feng Chen, Xiang Zhang, Jing-Hua Li, Lie Dai

**Affiliations:** Department of Rheumatology, Sun Yat-Sen Memorial Hospital, Sun Yat-Sen University, Guangzhou, 510120 People’s Republic of China; Department of Radiology, Sun Yat-Sen Memorial Hospital, Sun Yat-Sen University, Guangzhou, 510120 People’s Republic of China; Department of Anatomy and Developmental Biology, Monash University, Clayton, VIC 3800 Australia

## Abstract

**Introduction:**

Core disease activity indicators of rheumatoid arthritis (RA) have been found to be limited in predicting joint destruction progression. Matrix metalloproteinase (MMP) 3 plays an essential role in joint destruction and was found elevated in some remission patients. We aimed to monitor dynamic core disease activity indicators and serum MMP-3 for one year and evaluate their value for predicting radiographic progression.

**Methods:**

Patients with active RA (Simplified disease activity index > 3.3) were treated according to the treat-to-target strategy. Serum MMP-3 was detected by enzyme-linked immunosorbent assay and clinical data were collected simultaneously at 0, 1st, 3rd, 6th and 12th month. X-ray assessment of hand/wrist was repeated at baseline and the 12th month and a change of total Sharp score > 0.5 units was defined as radiographic progression.

**Results:**

Fifty-six patients completed one year follow-up and 29 % showed radiographic progression. Although not significantly different at baseline, serum MMP-3 and all core disease activity indicators, except for erythrocyte sedimentation rate, at the 12th month were significantly higher in the progressive group than in the non-progressive group. Among sixteen progressive patients, 69 % achieved the therapeutic target and 56 % had continuous elevated serum MMP-3, 38 % had continuous elevated serum MMP-3 and normal C-reactive protein (CRP) at the 6th month. Log-rank tests and repeated measures analysis revealed a significant difference in dynamic serum MMP-3 between progressive and non-progressive patients. Receiver operating characteristic curve and univariate logistic regression analysis showed that elevated serum MMP-3 at 0, 1st, 3rd and 6th months, compared with CRP at the 1st month, were significant predictors for one-year radiographic progression (MMP-3 odds ratio (*OR*):10.500 ~ 27.000, all *P* < 0.05; CRP: *OR* = 7.400, *P* = 0.011).

**Conclusions:**

Our data showed that continuously elevated serum MMP-3 for 3 ~ 6 months predicted one-year radiographic progression which implied that monitoring of dynamic serum MMP-3 combined with core disease activity indicators may be more helpful for predicting radiographic progression and treatment decision in RA.

## Introduction

Rheumatoid arthritis (RA) is a systemic autoimmune disease characterized by chronic synovitis and joint destruction which eventually lead to disability. It is a heterogeneous disease with a wide range of clinical manifestations from mild joint swelling with slowly progressive to severe polyarthritis with rapidly progressive destruction of cartilage and bone [1]. According to the current recommendations, RA treatment decision-making is mainly based on disease activity, and adjustment of therapy adheres to the treat-to-target (T2T) strategy which target is remission or low disease activity (LDA) [2]. However, a limitation of this strategy has been found in the prevention of structure destruction [3]. Radiographic progression has been reported in some remission RA patients, which implies the limitation of core disease activity indicators in predicting radiographic progression [4]. Considering the common disease activity indicators are unspecific for arthritis, novel biomarkers, such as inflammatory cytokines, destructive enzymes, breakdown products from collagenous and non-collagenous components of cartilage, and novel multi-biomarkers score containing several biomarkers, have been rapidly developed for predicting structural destruction progression in RA [5, 6].

Matrix metalloproteinase (MMP) 3 is a proteinase secreted by synovial fibroblasts and chondrocytes in joints. Active MMP-3 can accelerate joint destruction in RA by degrading aggrecan core protein, cartilage link protein, fibronectin, and collagen types IV, VII, IX and XI [7]. Serum MMP-3 had been well studied as an indicator of disease activity in RA. Our previous studies showed that serum MMP-3 level in RA was increased and positively correlated with disease activity, histological synovitis and synovial MMP-3 expression. However, some patients with remission or LDA also showed elevated serum MMP-3 and the outcome of these patients has been rarely reported [8, 9]. Considering the relationship between serum MMP-3 and joint destruction, we hypothesized that continuously elevated serum MMP-3 might be a predictor of radiographic progression. Here, we performed a prospective cohort study to monitor dynamic core disease indicators and serum MMP-3 for one year, and evaluated the value for predicting radiographic progression in RA.

## Materials and methods

### Patients

Patients with RA who fulfilled the 1987 revised criteria of the American College of Rheumatology (ACR) [10] or 2010 ACR/EULAR classification criteria for RA [11] were recruited from the Department of Rheumatology of Sun Yat-sen Memorial Hospital, Guangzhou, People’s Republic of China. Inclusion criteria also included: patients with active disease activity, defined as simplified disease activity index (SDAI) > 3.3 [12] and poor prognosis of one or more of the following features: functional limitation (Health Assessment Questionnaire score ≥ 1 [13]), positive rheumatoid factor (RF) or anti-cyclic citrullinated peptide antibody (anti-CCP), extraarticular disease (e.g., presence of rheumatoid nodules, secondary Sjogren’s syndrome, RA vasculitis, Felty’s syndrome, and RA lung disease) and bony erosions by radiography [14]. Exclusion criteria included: relevant concurrent liver disease (aspartate aminotransferase > 100 IU/L or alkalinephosphatase > 100 IU/L), renal disease (serum creatinine > 1.5 mg/dl), hematological disease (total white blood cell count < 4 × 10^9^/L, platelet count < 100 × 10^9^/L), or severe respiratory disease, malignancy, pregnancy or plans to become pregnant, and psychological problems that would make adherence to the study protocol impossible. This study was conducted in compliance with the Helsinki Declaration. The Medical Ethics Committee of Sun Yat-sen Memorial Hospital approved the protocol. All patients agreed to participate in this study and gave written informed consent.

### Treatment

All patients were treated according to the 2008 ACR recommendations for the management of RA [14]. Therapy was decided according to the disease duration, disease activity, presence of poor prognosis features and patients’ willingness. Disease duration was divided into three categories: < 6 months (short), 6–24 months (intermediate), and > 24 months (long) [14]. Disease activity defined by SDAI was divided into four categories: > 26.0 [high disease activity (HDA)], > 11.0 and ≤ 26.0 [moderate disease activity (MDA)], > 3.3 and ≤ 11.0 [low disease activity (LDA)], and ≤ 3.3 (remission) [12]. Adjustment of therapy was based on the T2T strategy adherence to the disease activity measured by SDAI. The therapeutic target was defined as remission (primary target) or LDA (alternative target) measured by SDAI and patients were divided into a T2T-achieving group (defined as SDAI remission + LDA at the 12th month) and a T2T-non-achieving group (MDA + HDA at the 12th month).

### Clinical assessments

All patients were followed up at regular intervals including the same predefined assessment points (0, 1st, 3rd, 6th, and 12th month) and the following indicators were assessed: 28-joint tender and swollen joint count (28TJC and 28SJC, both 0–28), Patient global assessment of disease activity (PtGA, 0–10 cm, 10 = worst status), Provider global assessment of disease activity (PrGA, 0–10 cm, 10 = worst status), Pain visual analogue scale (Pain VAS, 0–10 cm, 10 = most pain), Chinese language version of the Stanford health assessment questionnaire (HAQ, 0–3, 3 = most functional disability) [15], erythrocyte sedimentation rate [(ESR) mm/h, normal range: 0–20 mm/h (female), 0–15 mm/h (male)], C-reactive protein [(CRP), mg/dl, normal range: 0–0.5 mg/dl], RF (mg/L, determined by nephelometry, Siemens Healthcare Diagnostics, Munich, Germany, normal range: 0–20 mg/L), and anti-CCP [U/ml, measured by enzyme-linked immunosorbent assay (ELISA), Aesku Diagnostics, Wendelsheim, Germany, normal range: 0–18 U/ml]. Disease activity was assessed with SDAI, clinical disease activity index (CDAI) and disease activity score in 28 joints (DAS28) with four variables including CRP [DAS28 (4)-CRP].

### Radiographic assessments

Conventional radiographs of bilateral hands and wrists (anteroposterior view) were performed at baseline and the 12th month visit. All radiographs were scored according to the Sharp/van der Heijde score of hands by two experienced observers (MJD from rheumatology and ZX from radiology), who were not aware of the patients’ clinical findings [16]. Sixteen areas for erosion and fifteen for joint space narrowing of hands were assessed in each hand/wrist. The maximum score per single joint for erosions is 5, and for joint space narrowing is 4, with the sum of the erosion (0 ~ 160) and joint space narrowing (0 ~ 120) subscores constituting the total Sharp score of hands (0 ~ 280). Reliability and agreement were assessed using an intra-class correlation coefficient (ICC): the mean ICC for inter-observer agreement was 0.997. Radiographic progression was defined as a change of total Sharp score more than 0.5 units [17], and rapid radiographic progression (RRP) was defined as a change of total Sharp score more than 5 units from baseline to one year [18].

### Sandwich enzyme-linked immunosorbent assay for determination of serum MMP-3

Serum samples were collected from all the RA patients after overnight fasting and stored at −80 °C until analysis. Serum levels of soluble MMP-3 were measured with a human MMP-3 detection kit (AESKU Diagnostics, Wendelsheim, Germany) according to the manufacturer’s instructions. This kit detects total MMP-3 (pro- and active MMP-3) in human serum. Measurements were done in duplicate. Serum samples were placed in designated microwells. In addition, calibrators, negative and positive controls were added to the designated microwells to construct a standard curve. The plates were then incubated for 30 min at 26 °C and washed with wash buffer three times. Then 100 μl TMB substrate was added to each well and incubated for 30 min at 26 °C, protected from intense light. Then 100 μl of stop solution was added to each well, using the same order as for the substrate, and incubated for a minimum of 5 min. The absorbance of each well was read at 450 nm (optionally 450/620 nm) within 30 min. The normal ranges of serum MMP-3 concentrations were 18 ng/ml - 60 ng/ml (female) or 24 ng/ml – 120 ng/ml (male). The assays were performed blindly, without knowledge of the patient’s clinical data.

### Statistical analysis

Statistical analyses were performed with SPSS for Windows 13.0 statistical software (SPSS Inc., Chicago, IL, USA). Data are presented as frequencies and percentages for categorical variables and median and interquartile range (IQR) for continuous variables. The Mann–Whitney or Kruskal-Wallis rank-sum test was used to compare the differences of continuous variables between two or three groups. The Wilcoxon matched-pairs signed ranks sum test was used to compare the differences of continuous variables between indicators at baseline and each time point. The Chi-square test or Fisher exact test was used for categorical variables in different groups. Univariate and multivariate logistic regression analyses were used to identify predictors of radiological progression and control confounding factors. Variables were included in the model if *P* < 0.05 or removed if *P* > 0.10 according to the forward selection technique. The one-way analysis of variance (ANOVA) for repeated measures analysis with Bonferroni correction for multiple comparisons tests was used to compare the difference of dynamic disease activity indicators between two groups. Survival curves were used to show the ratio of patients with abnormal disease activity indicators during the therapies. Log-rank tests were used to compare survival curves of disease activity indicators between two groups. The abilities of disease activity indicators to predict one-year radiographic progression were evaluated by positive and negative predictive values (PPV and NPV, respectively). The predictive accuracy was assessed by receiver operating characteristic (ROC) curve analysis with area under the curve (AUC) and the cutoff point was determined by the Youden index. All significance tests were two-tailed and were conducted at the 5 % significance level.

## Results

### Demographic characteristics of RA patients at baseline

Sixty active RA patients who fulfilled the inclusion criteria were recruited from December 2010 to December 2013. Three patients were lost at the third month visit for migrating to other provinces and one patient was diagnosed with lung cancer four months after entry and excluded. Finally, fifty-six patients completed the study and were included for statistical analysis. Baseline demographic and clinical features of 56 patients with RA are shown in Table 1. The median of disease duration was 24 months ranging from 12 to 55 months. There were 84 % of patients who were RF positive and 82 % of patients who were anti-CCP positive. There were 46 % of patients without previous corticosteroid or disease-modifying antirheumatic drugs (DMARDS) therapy and 93 % of patients with bony erosions at baseline. There was no significant difference in the initial level of serum MMP-3 among patients with short, intermediate or long disease duration [247 (50 ~ 352) ng/ml vs 159 (63 ~ 291) ng/ml vs 279 (41 ~ 440) ng/ml, *P* = 0.875]. There was also no significant difference in the percentage of patients with elevated serum MMP-3 among short, intermediate or long disease duration groups (60 % vs 78 % vs 68 %, *P* = 0.593). There was no significant difference in the initial level of serum MMP-3 among patients with or without structural damage already present at baseline [137 (44 ~ 247) ng/ml vs 175 (63 ~ 380) ng/ml, *P* = 0.426].

**Table 1:** Baseline demographic and clinical features of RA patients

Characteristics	All patients (*n* = 56)	Non-progressive group (*n* = 40)	Progressive group (*n* = 16)	P*
Demographic				
Age, yrs, median (IQR)	48 (37 ~ 57)	48 (37 ~ 58)	51 (38 ~ 56)	0.863
Female, *n* (%)	43 (77)	33 (83)	10 (63)	0.211
Disease duration, mo, median (IQR)	24 (12 ~ 55)	17 (12 ~ 36)	24 (13 ~ 117)	0.154
Short duration, *n* (%)	5 (9)	3 (1)	2 (13)	0.941
Intermediate duration, *n* (%)	32 (57)	25 (63)	7 (44)	0.200
Long duration, *n* (%)	19 (34)	12 (30)	7 (44)	0.326
Core disease activity indicators				
ESR (mm/h), median (IQR)	56 (37 ~ 72)	56 (39 ~ 72)	57 (36 ~ 81)	0.856
CRP (mg/dl), median (IQR)	1.4 (0.7 ~ 3.3)	1.3 (0.6 ~ 3.0)	1.7 (0.9 ~ 5.5)	0.214
RF (mg/ml), median (IQR)	206 (61 ~ 475)	211 (42 ~ 465)	178 (69 ~ 510)	0.885
Anti-CCP (U/ml), median (IQR)	132 (32 ~ 300)	78 (26 ~ 300)	178 (53 ~ 425)	0.178
SDAI, median (IQR)	27.7 (16.0 ~ 39.5)	29.3 (14.2 ~ 42.6)	22.1 (16.3 ~ 36.1)	0.479
CDAI, median (IQR)	25.0 (14.3 ~ 36.8)	27.0 (13.0 ~ 39.0)	19.0 (15.3 ~ 32.8)	0.345
DAS28, median (IQR)	5.1 (3.8 ~ 5.9)	5.1 (3.9 ~ 6.1)	4.6 (3.9 ~ 5.7)	0.514
Poor prognosis features				
Function limitation, *n* (%)	30 (54)	20 (50)	10 (63)	0.397
RF positive, *n* (%)	47 (84)	32 (80)	15 (94)	0.388
Anti-CCP positive, *n* (%)	46 (82)	31 (78)	15 (94)	0.295
Extraarticular disease, *n* (%)	1 (2)^a^	0	1 (2)	NA
Bony erosions, *n* (%)	52 (93)	36 (90)	16 (100)	0.460
Total Sharp score, median (IQR)	11 (3 ~ 25)	8 (3 ~ 16)	22 (13 ~ 43)	*0.003*
Joint narrow score, median (IQR)	6 (1 ~ 13)	3 (1 ~ 8)	11 (6 ~ 25)	*0.014*
Erosion score, median (IQR)	5 (2 ~ 13)	4 (1 ~ 7)	13 (8 ~ 20)	*0.001*
Serum MMP-3 (ng/ml), median (IQR)	175 (57 ~ 365)	123 (45 ~ 276)	316 (195 ~ 563)	*0.010*
Hepatitis B virus infection, *n* (%)	5 (9)	2 (5)	3 (19)	0.135
Previous medications				
DMARDs and corticosteroids naïve, *n* (%)	26 (46)	21 (53)	5 (31)	0.253
Corticosteroids, *n* (%)	17 (30)	11 (28)	6 (38)	NA
Irregular, *n* (%)	9 (16)	5 (13)	4 (25)	NA
Prednisolone 10 mg/d, *n* (%)	4 (7)	4 (10)	0 (0)	NA
Prednisolone 5 mg/d, *n* (%)	4 (7)	2 (5)	2 (13)	NA
Methotrexate, *n* (%)	21 (38)	12 (30)	9 (56)	NA
Leflunomide, *n* (%)	10 (18)	7 (18)	3 (19)	NA
Sulfasalazine, *n* (%)	3 (5)	2 (5)	1 (6)	NA
Hydroxychloroquine, *n* (%)	7 (13)	3 (8)	4 (25)	NA
Cyclosporin A, *n* (%)	2 (4)	2 (5)	0	NA
Infliximab, *n* (%)	2 (4)	0	2 (12)	NA
YSP, *n* (%)	2 (4)	2 (5)	0	NA

*Compared between non-progressive group and progressive group by Mann–Whitney rank-sum test

*RA* rheumatoid arthritis, *CRP* C-reactive protein, *ESR* erythrocyte sedimentation rate, *MMP-3* matrix metalloproteinase-3, *RF* rheumatoid factor, *anti-CCP* anti-cyclic citrullinated peptide antibody, *DAS28* Disease Activity Score 28-joint assessment, *SDAI* simplified disease activity index, *CDAI* clinical disease activity index, *YSP* recombinant human tumor necrosis factor-α receptorII:IgG Fc fusion protein (Yi SaiPu), *IQR* interquartile range, *NA* not applicable

^a^RA vasculitis

### Clinical response and radiographic outcome

All RA patients had poor prognostic features. Thirty one (55 %) patients were treated with a combination of conventional synthetic DMARDs (csDMARDs) including methotrexate (MTX), leflunomide, hydroxychloroquine, sulfasalazine or cyclosporine A. Twenty five (45 %) patients were treated with a combination of csDMARD (mostly MTX) and biological DMARD [recombinant human tumor necrosis factor-α receptor-II (Yi Sai Pu, biosimilar) or infliximab]. Individualized adjustment of therapy was based on the T2T strategy and patient’s willingness. There were 91 % of patients treated with a combination of csDMARD while 9 % of patients were treated with a combination of csDMARD and TNF-α inhibitor at the 12th month. There were 7 %, 18 %, 39 %, and 34 % of patients who achieved SDAI remission, and 34 %, 41 %, 36 %, and 38 % of patients who achieved SDAI LDA at the 1st, 3rd, 6th, and 12th month, respectively (Table 2). At the 12th month, 16 (29 %) patients showed radiographic progression and five (9 %) patients had RRP. All patients were then divided into a progressive group (*n* = 16) and a non-progressive group (*n* = 40).

**Table 2:** Dynamic disease activity indicators and serum MMP-3 during one year follow-up

Disease activity indicators	1st month	3rd month	6th month	12th month
28TJC, median (IQR)	4 (1 ~ 9)***	2 (0 ~ 4)***	0 (0 ~ 4)***	1 (0 ~ 5)***
28SJC, median (IQR)	2 (0 ~ 4)***	0 (0 ~ 3)***	0 (0 ~ 1)***	0 (0 ~ 1)***
Pain VAS, median (IQR)	2.0 (1.7 ~ 4.0)***	2.0 (0 ~ 3.0)***	1.0 (0 ~ 2.8)***	1.0 (0 ~ 3.0)***
PtGA, median (IQR)	3.0 (3.0 ~ 5.0)***	3.0 (1.0 ~ 4.0)***	2.0 (0.3 ~ 3.0)***	2.0 (0 ~ 3.8)***
PrGA, median (IQR)	3.0 (2.3 ~ 4.0)***	2.0 (1.0 ~ 4.0)***	2.0 (0 ~ 3.0)***	2.0 (0 ~ 3.0)***
HAQ, median (IQR)	0.28 (0 ~ 0.94)***	0.15 (0 ~ 0.50)***	0 (0 ~ 0.25)***	0 (0 ~ 0.46)***
CRP, mg/dl, median (IQR)	0.44 (0.33 ~ 1.19)***	0.33 (0.30 ~ 0.95))***	0.34 (0.18 ~ 0.49)***	0.33 (0.30 ~ 0.89)***
Normal CRP^a^, %	54	59	77	70
ESR, mm/h, median (IQR)	34 (18 ~ 48)***	29 (16 ~ 38)***	21 (15 ~ 36)***	26 (13 ~ 42)***
Norma ESR^b^, %	27	38	41	39
DAS28^c^, median (IQR)	3.5 (2.6 ~ 4.4)***	2.7 (2.0 ~ 3.9)***	2.2 (1.5 ~ 3.2)***	2.6 (1.5 ~ 3.3)***
Remission, %	27	43	53	48
LDA, %	20	20	20	21
MDA, %	44	30	20	26
HDA, %	9	7	7	5
SDAI^d^, median (IQR)	14.5 (7.6 ~ 24.2)***	8.9 (4.1 ~ 17.5)***	5.3 (1.0 ~ 12.0)***	6.4 (0.3 ~ 12.3)***
Remission, %	7	18	39	34
LDA, %	34	41	36	38
MDA, %	38	30	16	19
HDA, %	21	11	9	9
CDAI^e^, median (IQR)	13.7 (7.1 ~ 22.8)***	8.0 (4.0 ~ 15.9)***	4.5 (1.0 ~ 11.5)***	5.5 (0 ~ 11.0)***
Remission, %	7	18	39	38
LDA, %	34	45	36	36
MDA, %	34	26	16	16
HDA, %	25	11	9	11
Serum MMP-3, ng/ml, median (IQR)	112 (49 ~ 347)	130 (32 ~ 183)	74 (32 ~ 183)*	52 (32 ~ 116)***
Normal serum MMP-3^f^, %	32	38	46	62
RF, mg/ml, median (IQR)	119 (39 ~ 252)***	72 (20 ~ 169)***	49 (14 ~ 137)***	78(16 ~ 142)***
Anti-CCP, U/ml, median (IQR)	85 (11 ~ 300)**	53 (13 ~ 296)***	65 (16 ~ 297)**	112(14 ~ 300)*

*Pain VAS* pain visual analogue scale, *28TJC* 28-joint tender joint count, *28SJC* 28-joint swollen joint count, *PtGA* patient global assessment of disease activity, *PrGA* provider global assessment of disease activity, *HAQ* health assessment questionnaire, *CRP* C-reactive protein, *ESR* erythrocyte sedimentation rate, *DAS28* Disease Activity Score 28-joint assessment, *SDAI* simplified disease activity index, *CDAI* clinical disease activity index, *LDA* low disease activity, *MDA* moderate disease activity, *HDA* high disease activity, *MMP-3* matrix metalloproteinase-3, *RF* rheumatoid factor, *anti-CCP* anti-cyclic citrullinated peptide antibody

*P < 0.05, **P < 0.01, ***P < 0.001. Compared between each time point and baseline by Wilcoxon matched-pairs signed ranks sum test

^a^normal CRP: <0.5 mg/dl; ^b^normal ESR: <20 mm/h (female) or <15 mm/h (male); ^c^DAS28-remission: <2.6; DAS28-LDA:2.6 ~ 3.2, DAS28-MDA:3.2 ~ 5.1, DAS28-HDA:>5.1; ^d^SDAI-remission: <3.3; SDAI-LDA: 3.3 ~ 11.0, SDAI-MDA: 11.0 ~ 26.0, SDAI-HDA: >26.0; ^e^CDAI-remission: <2.8; CDAI-LDA: 2.8 ~ 10.0, CDAI-MDA: 10.0 ~ 22.0, CDAI-HDA: >22.0; ^f^normal serum MMP-3: <60 ng/ml (female) or <120 ng/ml (male)

### Dynamic change of disease activity indicators and serum MMP-3

**Fig. 1:**
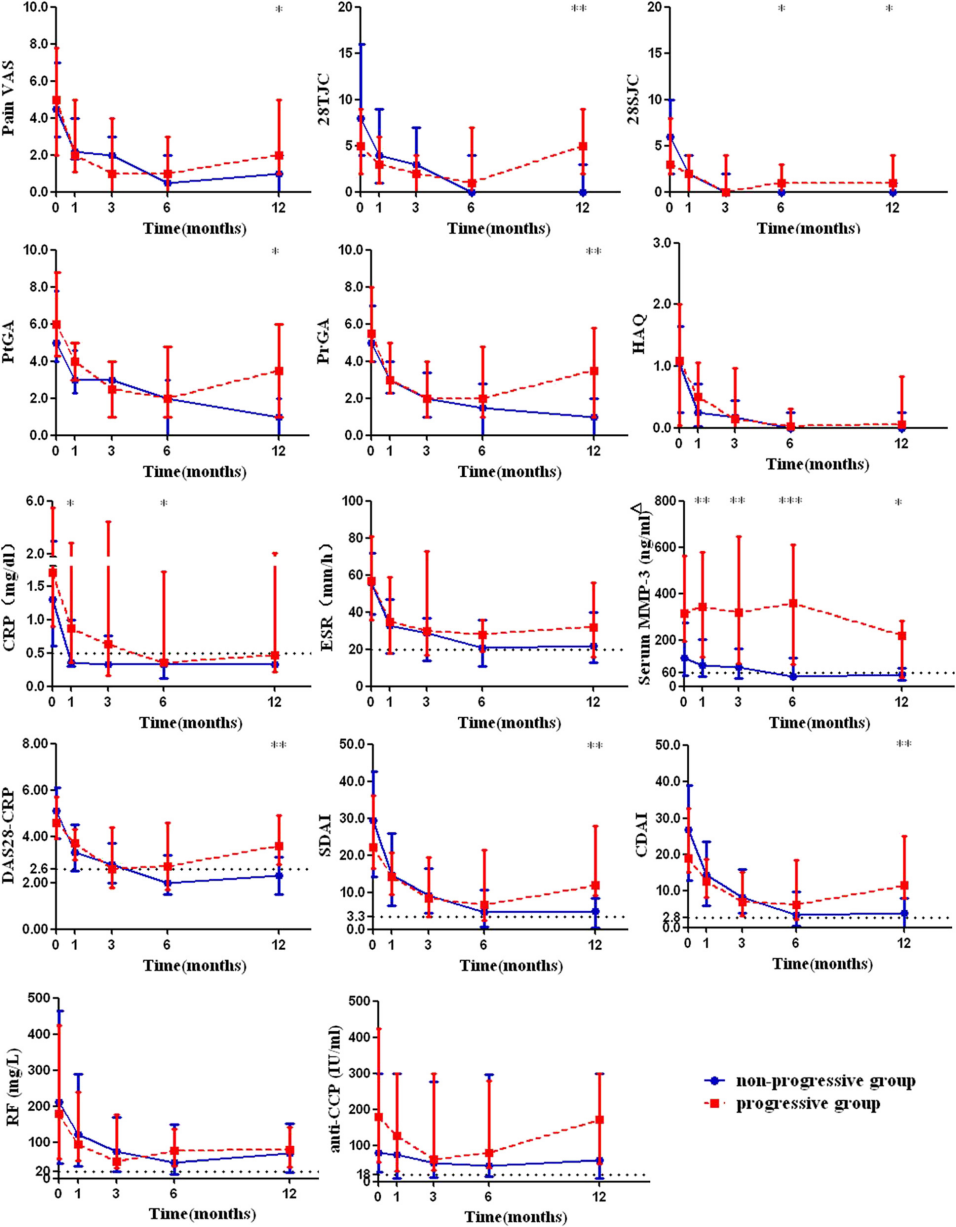
Dynamic disease activity indicators and serum MMP-3 between non-progressive and progressive patients. *P < 0.05, **P < 0.01, ***P < 0.001. Compared between non-progressive and progressive groups by Mann–Whitney rank-sum test. White triangle: dotted line of 60 represents the upper limit of normal serum MMP-3 in females (upper limit of normal serum MMP-3 in males is not shown); *Pain VAS* pain visual analogue scale, *28TJC* 28-joint tender joint count, *28SJC* 28-joint swollen joint count, *PtGA* patient global assessment of disease activity, *PrGA* provider global assessment of disease activity, *HAQ* health assessment questionnaire, *CRP* C-reactive protein, *ESR* erythrocyte sedimentation rate, *DAS28* Disease Activity Score 28-joint assessment, *SDAI* simplified disease activity index, *CDAI* clinical disease activity index, *MMP-3* matrix metalloproteinase-3, *RF* rheumatoid factor, *anti-CCP* anti-cyclic citrullinated peptide antibody. Data are represented by median and interquartile range

The results of dynamic disease activity indicators and serum MMP-3 during one year follow-up are shown in Table 2 and Fig. 1. Although there was no significant difference at baseline, all core disease activity indicators except for ESR at the 12th month were significantly higher in the progressive group than in the non-progressive group (all *P* < 0.05). Among sixteen patients with radiographic progression, eleven (69 %) of them had achieved the therapeutic target at the 6th month and nine (56 %) patients had continuously elevated serum MMP-3, six (38 %) patients had continuously elevated serum MMP-3 and normal CRP at the 6th month. There were 82 % of the RA patients with elevated CRP and 73 % with elevated serum MMP-3 at baseline and CRP decreased more quickly than serum MMP-3 after treatment. There were 54 %, 59 %, 77 %, and 70 % of RA patients with normal CRP at the 1st, 3rd, 6th, and 12th month, respectively, compared with 32 %, 38 %, 46 %, and 62 % of RA patients with normal serum MMP-3 at the same time points.

**Fig. 2:**
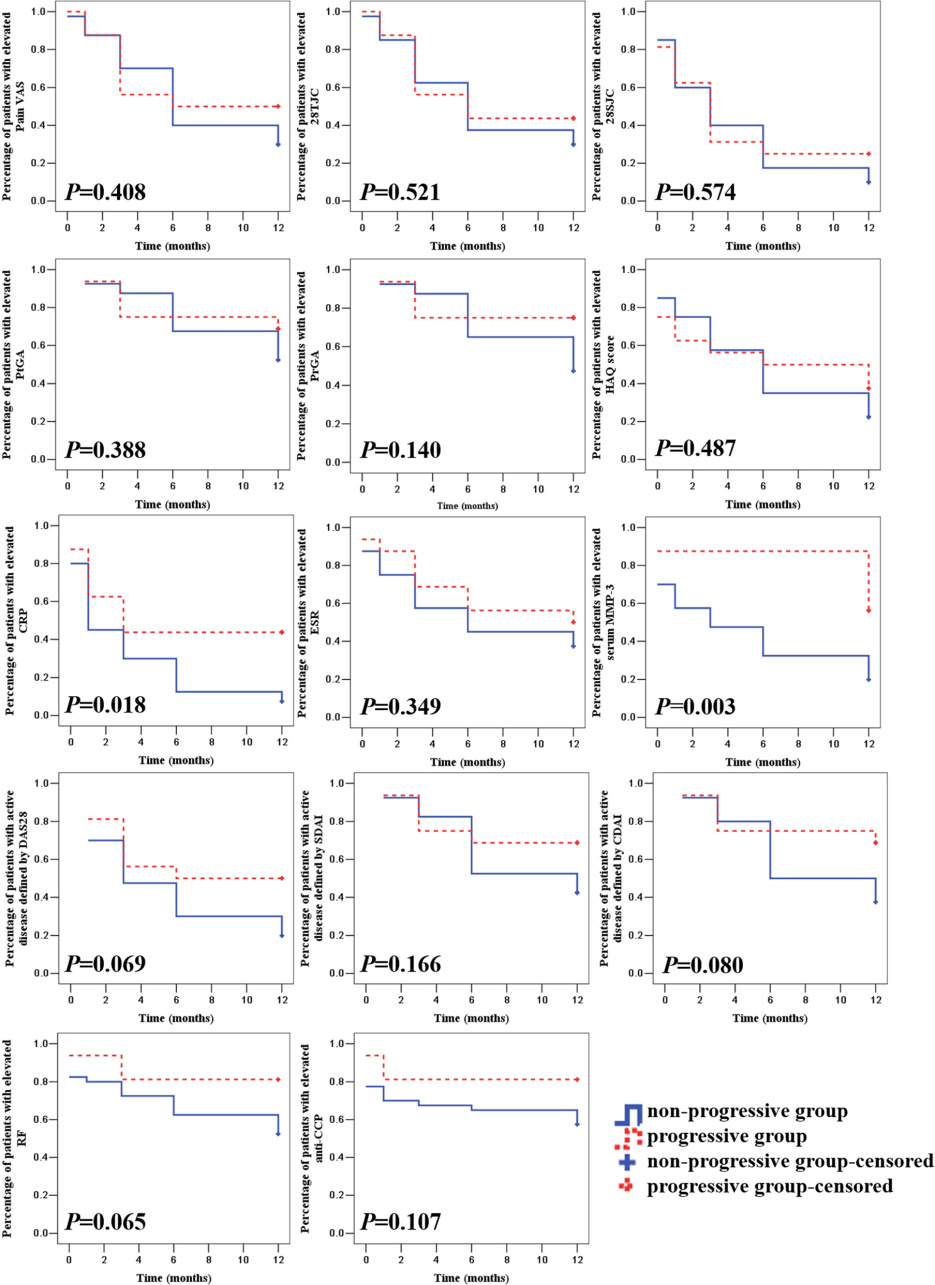
Survival curves for the ratio of patients with abnormal disease activity indicators and serum MMP-3 between non-progressive and progressive patients. *P* values were determined by log-rank tests between non-progressive and progressive groups. *Pain VAS* pain visual analogue scale, *28TJC* 28-joint tender joint count, *28SJC* 28-joint swollen joint count, *PtGA* patient global assessment of disease activity, *PrGA* provider global assessment of disease activity, *HAQ* health assessment questionnaire, *CRP* C-reactive protein, *ESR* erythrocyte sedimentation rate, *DAS28* Disease Activity Score 28-joint assessment, *SDAI* simplified disease activity index, *CDAI* clinical disease activity index, *MMP-3* matrix metalloproteinase-3, *RF* rheumatoid factor, *anti-CCP* anti-cyclic citrullinated peptide antibody

Further analysis showed 38 %, 50 %, and 56 % of progressive patients with normal CRP at the first, third, and sixth months, respectively, compared with 87 % of progressive patients with continuously elevated serum MMP-3 for more than six months (data not shown). Serum MMP-3 was significantly higher in the progressive group than in the non-progressive group at baseline and the 1st, 3rd, 6th, and 12th months (all *P* < 0.05). Survival curves were used to show the ratio of patients with elevated disease activity indicators during follow-up and log-rank tests revealed significant differences in the survival curves of patients with elevated serum MMP-3 and CRP between progressive and non-progressive groups (both *P* < 0.05, Fig. 2). The ANOVA for repeated measures analysis and multiple comparisons tests further confirmed the difference in dynamic serum MMP-3 between these two groups (data not shown).

### Predictors of one-year radiographic progression

Among the baseline characteristics, serum MMP-3, total Sharp score, joint narrow score and erosion score were significantly higher in the progressive group than in the non-progressive group (all *P* < 0.05, Table 1), while there was no significant difference in age, sex, core disease activity indicators, functional limitation, RF or anti-CCP positive rate, or the percentage of DMARD- or corticosteroid-naïve patients at baseline between progressive and non-progressive groups (all *P* > 0.05). ROC curve analysis showed that the predictive accuracy of serum MMP-3 for one-year radiographic progression was 0.721 with a cutoff point of 159 ng/ml (*P* = 0.010, PPV 46.7 % and NPV 92.3 %), and the predictive accuracy of total Sharp score was 0.753 with a cutoff point of 15 (*P* = 0.003, PPV 54.5 % and NPV 88.2 %). Univariate logistic regression showed that elevated serum MMP-3 (>159 ng/ml) and total Sharp score (>15) at baseline were significant predictors of one-year radiographic progression (all *P* < 0.05, Table 3). Multivariate logistic regression analysis was performed to control confounding factors which included all the baseline parameters mentioned above and showed that high serum MMP-3 (>159 ng/ml) and high total Sharp score (>15) were included in the final equation while other parameters were excluded (data not shown).

**Table 3:** Performance of disease activity indicators as predictors for one-year radiographic progression^a^

Disease activity indicators	Accuracy	*P*	95 % *CI*	Cutoff point	PPV (%)	NPV (%)	*OR* (95 % *CI*)	*P*
Baseline								
Serum MMP-3	0.721	0.010	0.572 ~ 0.870	159 ng/ml	46.7	92.3	10.500 (2.097 ~ 52.581)	0.004
Total Sharp score	0.753	0.003	0.613 ~ 0.893	15	54.5	88.2	9.000 (2.359 ~ 34.334)	0.001
1st month								
CRP	0.680	0.037	0.525 ~ 0.835	1.76 mg/dl	66.7	78.7	7.400 (1.567 ~ 34.935)	0.011
Serum MMP-3	0.758	0.003	0.605 ~ 0.911	264 ng/ml	64.7	87.2	12.467 (3.175 ~ 48.950)	< 0.001
3rd month								
Serum MMP-3	0.766	0.002	0.606 ~ 0.926	178 ng/ml	64.7	87.2	15.857 (3.412 ~ 73.687)	< 0.001
6th month								
28SJC	0.667	0.052	0.503 ~ 0.832	NA	NA	NA	NA	NA
CRP	0.681	0.035	0.525 ~ 0.838	0.64 mg/dl	58.3	79.5	5.444 (1.395 ~ 21.244)	0.015
Serum MMP-3	0.830	< 0.001	0.696 ~ 0.963	161 ng/ml	75.0	90.0	27.000 (5.834 ~ 24.962)	< 0.001

^a^Accuracy of predictive value was determined by ROC curve analysis; OR of predictors was determined by univariate logistic regression analysis according to the cutoff point

*28SJC* 28-joint swollen joint count, *PrGA* provider global assessment of disease activity, *CRP* C-reactive protein, *ESR* erythrocyte sedimentation rate, *MMP-3* matrix metalloproteinase-3, *RF* rheumatoid factor, *anti-CCP* anti-cyclic citrullinated peptide antibody, *DAS28* Disease Activity Score 28-joint assessment, *SDAI* simplified disease activity index, *PPV* positive predictive value, *NPV* negative predictive value, *ROC* receiver operating characteristic; *OR* odds ratio, *CI* confidence interval, *NA* not applicable

At the first month follow-up, CRP and serum MMP-3 were significantly higher in the progressive group than in non-progressive group (all *P* < 0.05, Fig. 1), while other disease activity indicators showed no significant differences between these two groups (all *P* > 0.05). ROC curve analysis showed that the predictive accuracy of CRP for one-year radiographic progression was 0.680 with a cutoff point of 1.76 mg/dl (P = 0.037, PPV 66.7 % and NPV 78.7 %), and serum MMP-3 was 0.758 with a cutoff point of 264 ng/ml (P = 0.003, PPV 64.7 % and NPV 87.2 %). Univariate logistic regression showed that CRP (>1.76 mg/dl) and serum MMP-3 (>264 ng/ml) at the first month were significant predictors of one-year radiographic progression (all *P* < 0.05, Table 3).

At the third month follow-up, serum MMP-3 was significantly higher in the progressive group than that in the non-progressive group (*P =* 0.002, Fig. 1). ROC curve analysis showed that the predictive accuracy of serum MMP-3 for one-year radiographic progression was 0.766 with a cutoff point of 178 ng/ml (*P* < 0.001, PPV 64.7 % and NPV 87.2 %). Univariate logistic regression showed that serum MMP-3 at the third month (>178 ng/ml) was a significant predictor of one-year radiographic progression (all *P* < 0.01, Table 3).

At the six month follow-up, 28SJC, CRP, and serum MMP-3 were significantly higher in the progressive group than in the non-progressive group (all *P* < 0.05, Fig. 1). ROC curve analysis showed that the predictive accuracy of CRP for one-year radiographic progression was 0.681 with a cutoff point of 0.64 mg/dl (*P* = 0.035, PPV 58.3 % and NPV 79.5 %), and serum MMP-3 was 0.830 with a cutoff point of 161 ng/ml (*P* < 0.001, PPV 75.0 % and NPV 90.0 %). Univariate logistic regression showed that CRP (>0.64 mg/dl) and serum MMP-3 (>161 ng/ml) at the sixth month were significant predictors of one-year radiographic progression (all *P* < 0.05, Table 3).

### Dynamic serum MMP-3 and T2T strategy

**Fig. 3:**
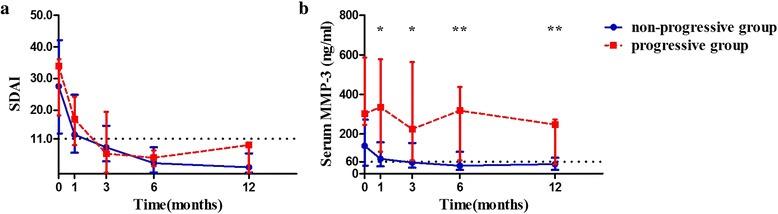
Dynamic disease activity and serum MMP-3 between non-progressive and progressive patients in T2T-achieving group (**a**. Dynamic disease activity defined by SDAI; **b**. Dynamic serum MMP-3). *P < 0.05, **P < 0.01. Compared between non-progressive and progressive groups by Mann–Whitney rank-sum test. White triangle: dotted line of 60 represents the upper limit of normal serum MMP-3 in females (upper limit of normal serum MMP-3 in males is not shown). *SDAI* simplified disease activity index, *MMP-3* matrix metalloproteinase-3. Data are represented by median and interquartile ranges

There were 40 (71 %) patients who achieved the therapeutic target at the 12th month. In the T2T-achieving group, seven (18 %) patients developed radiographic progression, of which six (86 %) showed elevated serum MMP-3 at baseline, first, third, and sixth months. There was no significant difference of disease activity (measured by SDAI) between progressive and non-progressive patients in the T2T-achieving group throughout one year, while serum MMP-3 was significantly higher in progressive patients than in non-progressive patients at baseline, 1st, 3rd, 6th or 12th month (all *P* < 0.05, Fig. 3). ROC curve analysis showed that the predictive accuracy of serum MMP-3 at baseline, first, third and sixth month for one-year rapid radiographic progression was 0.745, 0.801, 0.784, and 0.840, respectively, with cutoff points of 242, 297, 193, and 161 ng/ml, respectively (all *P* < 0.05). Univariate logistic regression showed that elevated serum MMP-3s (> cutoff points) at baseline and first, third and sixth month were significant predictors of one-year rapid radiographic progression (Table 4). In the T2T non-achieved group (*n* = 16, 29 %), nine (56 %) developed radiographic progression, of which eight (89 %) showed elevated serum MMP-3 at the sixth month.

**Table 4:** Performance of dynamic serum MMP-3 as predictor for one-year radiographic progression^a^

Serum MMP-3	Accuracy	*P*	95 % *CI*	Cutoff point	PPV (%)	NPV (%)	*OR* (95 % *CI*)	*P*
Baseline	0.745	0.044	0.564 ~ 0.925	242 ng/ml	37.5	95.8	13.800 (1.464 ~ 130.070)	0.022
1st month	0.801	0.013	0.606 ~ 0.996	297 ng/ml	55.6	93.5	18.125 (2.592 ~ 126.721)	0.003
3rd month	0.784	0.020	0.564 ~ 1.003	193 ng/ml	55.6	93.5	18.125 (2.592 ~ 126.721)	0.003
6th month	0.840	0.005	0.658 ~ 1.022	161 ng/ml	62.5	93.8	25.000 (3.302 ~ 189.259)	0.002

^a^Accuracy of predictive value was determined by ROC curve analysis; ORs of predictors were determined by univariate logistic regression analysis according to the cutoff point

*MMP-3* matrix metalloproteinase-3, *PPV* positive predictive value, *NPV* negative predictive value, *ROC* receiver operating characteristic, *OR* odds ratio, *CI* confidence interval

There were five patients with LDA at baseline and four of them achieved remission at the 6th month without radiographic progression at the 12th month; meanwhile, their serum MMP-3 remained normal from baseline to the 6th month. The other patient with LDA (SDAI = 8.9) at baseline became worse with MDA (SDAI = 24.3) and developed radiographic progression at the 12th month together with continuously elevated serum MMP-3 throughout the whole year.

### Patients with RRP after one year

There were five patients with RRP at the one-year follow-up, of which four patients did not achieve the therapeutic target. Baseline serum MMP-3 and total Sharp score were significantly higher in patients with RRP than in other patients (both *P* < 0.05), while there was no significant difference in age, disease duration, other core disease activity indicators, functional limitation, RF or anti-CCP positive rate between patients with or without RRP (all *P* > 0.05). Baseline SDAI of these patients with RRP was 20.5 (16.0 ~ 30.8). One patient with RRP achieved LDA at the first month and remained there for the entire year, while the others failed to achieve the therapeutic target during one-year follow-up. Serum MMP-3 at the third month and 28SJC, PtGA, PrGA, and SDAI at the sixth month were significantly higher in patients with RRP than in other patients (all *P* < 0.05). ROC curve analysis showed that the predictive accuracy of serum MMP-3 at the third month for one-year RRP was 0.784 with a cutoff point of 178 ng/ml (*P* = 0.037, PPV 23.5 % and NPV 97.4 %). Univariate logistic regression showed that serum MMP-3 (>178 ng/ml) at the third month was a significant predictor of one-year RRP (odds ratio (*OR*) = 11.692, 95 % confidence interval (*CI*): 1.196 ~ 114.312, *P* = 0.035).

## Discussion

This research performed a prospective cohort study comparing serum MMP-3 with core disease activity indicators for predicting radiographic progression in RA. Dynamic serum MMP-3 and core disease activity indicators were analyzed by repeated measures analysis and log-rank tests. The results showed continuously elevated serum MMP-3 for 3 ~ 6 months in radiographic progressive patients who have achieved the therapeutic target even when combined with normal CRP. Further analyses of ROC curve and univariate logistic regression analysis showed that elevated serum MMP-3 at baseline and the first, third, and sixth month, compared with CRP only at the first month, were significant predictors for one-year radiographic progression in RA. Subgroup studies showed that dynamic serum MMP-3 might be especially helpful for predicting radiographic progression in the T2T-achieving group, and elevated serum MMP-3 at the third month was a significant predictor of one-year RRP in patients with RRP.

Joint destruction in RA may reduce the quality of life and cause severe disability. Conventional X-ray radiography is the most commonly used method to reflect existing joint damage. Several scoring systems, such as the Larsen and Sharp score systems, have been developed for quantitative evaluation. The Sharp score system was established for the assessment of hands in 1971 and modified by van der Heijde in 1985 who optimized the scoring procedures and added the assessment of feet [16]. The Sharp/van der Heijde score system which is sensitive for detecting radiographic change has been widely used in clinical research of RA [19].

The Sharp/van der Heijde score can be summed in several ways: total erosion of hands and/or feet; total joint space narrowing of hands and/or feet; and summation of erosions and joint space narrowing, called “total score” of hands and/or feet [16]. Metacarpophalangeal (MCP) joints, proximal interphalangeal (PIP) joints and wrists are the most commonly involved joints in RA (78 % ~ 91 %), while foot involvement appeared in less than half of RA patients (43 %) [20]. We applied the Sharp/van der Heijde score containing MCP, PIP and wrists in this study.

Previous studies have confirmed a significant correlation between disease activity and joint damage. Both ACR and EULAR recommendations on treatment of RA emphasize tight control of disease activity and therapeutic target of remission or LDA [2, 21]. Our study was designed as a real world prospective cohort study using the T2T strategy. The therapeutic target was achieved at the 12th month by 71 % of the patients which is comparable with published rates of clinical studies (31 % ~ 75 %) [22–26]. CRP significantly decreased after one month and returned to normal after three and six months in 59 % and 77 % of the patients, respectively. However, 29 % of the patients showed radiographic progression after one year, and most core disease activity indicators except for ESR at the 12th month were significantly higher in the progressive group than in the non-progressive groups although there was no significant difference at baseline. To exclude the joint damage progression due to not being able to be intensively treated, we performed a subgroup analysis of T2T-achieving patients and found that seven (18 %) of them had radiographic progression. These results indicate the limitation of the T2T strategy focused on disease activity in the prevention of joint destruction and the limitation of dynamic CRP in predicting radiographic progression. A new biomarker for predicting radiographic progression in RA is needed.

As a proteinase from local joints, MMP-3 can be detected in peripheral blood. MMP-3 was first measured by a substrate cleavage assay or zymography at 1986. However, the substrate cleavage assay lacked convenience, specificity or sensitivity, while zymography was labor-intensive and semi-quantitative. The assay methodology was modified to ELISA using horseradish peroxidase (HRP) as the detection enzyme and colorimetric signal in 1992. The specificity of MMP-3 detection is very important as other MMPs, such as MMP-1, MMP-2 and MMP-9, have a similar structure and same origin which are produced at the same site or by the same cells [27]. Here, we used the AESKULISA DF MMP-3 kit to detect serum MMP-3 by sandwich ELISA using microplates coated with monoclonal anti-human MMP-3 antibody and no cross-reactivity to other antigens including MMP-1, MMP-2 or MMP-9 had been found according to the performance data of this kit [8].

Previous studies have found that baseline serum MMP-3 is positively correlated with disease activity and joint destruction at entry and it can predict radiographic progression longitudinally in early RA [28]. In this study, we also found that high serum MMP-3 and high total Sharp score at baseline could predict one year radiographic progression. Compared to radiographic assessment, especially X-ray which cannot be performed very frequently, serum MMP-3 might be more suitable for dynamic monitoring with an interval as short as 1 ~ 3 months. Our dynamic data showed that serum MMP-3 is significantly higher in progressive patients than in non-progressive patients for an entire year and 87 % of progressive patients showed continuously elevated serum MMP-3 for more than six months, compared with 44 % of progressive patients with elevated CRP after six months. ROC curve and univariate logistic regression analyses showed that serum MMP-3 at baseline and the first, third, and sixth month were significant predictors of one-year radiographic progression. A recent systematic literature review of 57 clinical studies also found that time-integrated measure of disease activity indicators positively correlated with radiographic progression which implies that monitoring of dynamic disease activity indicators might be more valuable in predicting radiographic progression [29]. All these findings indicate that dynamic monitoring of serum MMP-3 may be helpful for predicting radiographic progression in RA and continuously elevated serum MMP-3 for 3 ~ 6 months may be a significant predictor of one-year radiographic progression.

Young-Min et al. reported elevated baseline serum MMP-3 as a predictor of two-year radiographic progression with a cutoff point of 85.79 ng/ml and also a strong independent predictor of radiographic progression at 8.2 years with a cutoff point of 71.6 ng/ml, in which the cutoff points of serum MMP-3 were within the normal range of male (17.3 ~ 59.7 ng/ml in female and 36.9 ~ 121 ng/ml in male, Daiichi Fine Chemical, Toyama, Japan) [30, 31]. Here we found that elevated serum MMP-3 at baseline and the first, third and sixth months were significant predictors of one-year radiographic progression with cutoff points of 159 ng/ml, 264 ng/ml, 178 ng/ml, and 161 ng/ml, respectively. Considering the close relationship of sensitivity, specificity and PPV, NPV, we performed ROC curve analysis with Youden index to determine the cutoff points and found that the cutoff points in both female and male patients (such as 161 ng/ml at the 6th month) were higher than the upper limit of the normal range which will be more applicable for clinical use.

The reduction of serum MMP-3 was recently considered as a secondary therapeutic target, particularly in biological therapy with TNF-α inhibitor, IL-6 receptor blocker or T-cell costimulation inhibitor therapies [32–34]. Urata et al. reported a study of 243 early RA patients who were divided into routine care, DAS28 driven therapy, MMP-3 driven therapy or both DAS28 and MMP-3 driven therapy groups and the result of one-year follow-up showed that the MMP-3 driven therapy group had the greatest proportion to achieve radiographic non-progression among these four groups, which implied that serum MMP-3 might be a supplemental predictor of radiographic progression in clinical practice [25]. According to ACR/EULAR recommendations on treatment of RA, the therapeutic target should be achieved in 3 ~ 6 months [2, 14, 21]. In our data, there were nine (16 %) patients with continuous elevated serum MMP3 at 3 ~ 6 months who had achieved the therapeutic target at that time, but finally developed radiographic progression. If these patients were treated more aggressively until serum MMP-3 returned to normal, the possibility of radiographic progression after one year might be decreased. The combined strategy of both SDAI or CDAI and serum MMP-3 driven therapy is worth further study.

There are several limitations of this study. Firstly, it was designed as a real world prospective cohort study. All patients were recruited at a single center and treated with various medications. Further multicenter studies of the combined strategy of both disease activity and serum MMP-3 driven therapy with the same treatment in all centers are needed. Secondly, all patients recruited in this study had poor prognostic features and 93 % of them had bony erosion at baseline which might confound prediction of radiographic progression. More new onset RA patients without bony erosion are needed in future to investigate whether serum MMP-3 could predict radiographic progression in these patients.

## Conclusions

Our data show that continuously elevated serum MMP-3 for 3 ~ 6 months predicted one-year radiographic progression which implies that monitoring of dynamic serum MMP-3 combined with core disease activity indicators may be more helpful for predicting radiographic progression and treatment decisions in RA.

## Abbreviations

28SJC: 28-joint swollen joint count
28TJC: 28-joint tender joint count
anti-CCP: Anti-cyclic citrullinated peptide antibody
AUC: Area under the curve
CDAI: Clinical disease activity index
CI: Confidence interval
CRP: C-reactive protein
DAS28: Disease Activity Score 28-joint assessment
ESR: Erythrocyte sedimentation rate
HAQ: Chinese language version of Stanford Health Assessment Questionnaire
HDA: High disease activity
IQR: Interquartile range
LDA: Low disease activity
MDA: Moderate disease activity
MMP-3: Matrix metalloproteinase-3
NPV: Negative predictive value
OR: Odds ratio
Pain VAS: Pain visual analogue scale
PPV: Positive predictive value
PrGA: Provider global assessment of disease activity
PtGA: Patient global assessment of disease activity
RA: Rheumatoid arthritis
RF: Rheumatoid factor
ROC curve: Receiver operating characteristic curve
RRP: Rapid radiographic progression
SDAI: Simplified disease activity index
TNF-α: Tumor necrosis factor alpha
YSP: Recombinant human tumor necrosis factor-α receptorII:IgG Fc fusion protein (Yi Sai Pu)
