# Erratum to: Continuously elevated serum matrix metalloproteinase-3 for 3 ~ 6 months predict one-year radiographic progression in rheumatoid arthritis: a prospective cohort study

**DOI:** 10.1186/s13075-015-0837-5

**Published:** 2015-11-04

**Authors:** Jian-Da Ma, Xiu-Ning Wei, Dong-Hui Zheng, Ying-Qian Mo, Le-Feng Chen, Xiang Zhang, Jin-Hua Li, Lie Dai

**Affiliations:** Department of Rheumatology, Sun Yat-Sen Memorial Hospital, Sun Yat-Sen University, Guangzhou, 510120 People’s Republic of China; Department of Radiology, Sun Yat-Sen Memorial Hospital, Sun Yat-Sen University, Guangzhou, 510120 People’s Republic of China; Department of Anatomy and Developmental Biology, Monash University, Clayton, 3800 VIC Australia

Unfortunately, the original version of this article [1] contained an error. The author name ‘Jing-Hua Li’ was included incorrectly. This should be spelt ‘Jin-Hua Li’ as corrected in the author list above. The author name has been corrected in the original article also.
